# The prevalence and risk factors for acute respiratory infections in children aged 0‐59 months in rural Malawi: A cross‐sectional study

**DOI:** 10.1111/irv.12481

**Published:** 2017-09-30

**Authors:** Miriam Cox, Louis Rose, Khumbo Kalua, Gilles de Wildt, Robin Bailey, John Hart

**Affiliations:** ^1^ School of Clinical and Experimental Medicine College of Medicine and Dental Sciences University of Birmingham Birmingham UK; ^2^ Department of Ophthalmology College of Medicine University of Malawi Blantyre Malawi; ^3^ Department of Clinical Research London School of Hygiene and Tropical Medicine London UK

**Keywords:** acute respiratory infection, Malawi, malnutrition, preschool children

## Abstract

**Background:**

Acute Respiratory Infections (ARI) are a leading cause of childhood mortality and morbidity. Malawi has high childhood mortality but limited data on the prevalence of disease in the community.

**Methods:**

A cross‐sectional study of children aged 0‐59 months. Health passports were examined for ARI diagnoses in the preceding 12 months. Children were physically examined for malnutrition or current ARI.

**Results:**

828 children participated. The annual prevalence of ARI was 32.6% (95% CI 29.3‐36.0%). Having a sibling with ARI (OR 1.44, *P *= .01), increasing household density (OR 2.17, *P *=* *.02) and acute malnutrition (OR 1.69, *P *=* *.01) were predictors of infection in the last year. The point prevalence of ARI was 8.3% (95% CI 6.8‐10.4%). Risk factors for current ARI were acute‐on‐chronic malnutrition (OR 3.06, *P *=* *.02), increasing household density (OR1.19, *P *=* *.05) and having a sibling with ARI (OR 2.30, *P *=* *.02).

**Conclusion:**

This study provides novel data on the high prevalence of ARI in Malawi. This baseline data can be used in the monitoring and planning of future interventions in this population.

## 1 INTRODUCTION

Global reduction in childhood mortality by 66% is a millennium development goal.^1^ Acute respiratory infections (ARIs) encompass bacterial and viral infections of the upper respiratory tract (URT) or lower respiratory tract (LRI). ARI are the commonest cause of illness and mortality in under 5s resulting in over 900,000 deaths annually, most of which are due to pneumonia.^2^ Malawi, the world's poorest country,^3^ has an under 5 mortality of 64/1000.^4^ Over 70% of Malawian children seek treatment for ARIs,^4^ and they cause 6%‐40% of childhood mortality.^5^, ^6^ Children living rurally are more likely to be affected,^6^^.^ 7 The prevalence of respiratory tract infections in the community has not been studied in rural areas of Malawi; the burden of disease to healthcare services is unknown.

Risk factors for mortality from childhood acute respiratory infection (ARI) in Malawi include HIV coinfection, acute malnutrition preterm birth and household overcrowding,^8^, ^9^, ^10^, ^11^, ^12^, ^13^ estimating mortality in hospitalized children at 2.0%‐5.6%.^12^, ^13^ Therefore, research determining the generalizability of these risk factors to children in the community was needed. The 13‐valent pneumococcal conjugate vaccination (PCV13) was introduced in Malawi in November 2011 and has been shown to decrease severe childhood pneumonia by 65% in both hospitalized and community cases.^14^ Malawi has a national programme for PCV, and recent data suggest that 49.9% of children under five receive PCV, but the uptake in Mangochi is unknown. Malawi's Expanded Programme of Immunization (EPI) includes tuberculosis, polio, tetanus, haemophilus influenzae B, measles and rotavirus.^15^ Approximately 80% of Malawian children receive EPI immunizations with uptake being lower in rural communities.^16^, ^17^, ^18^ Regional variations in vaccination uptake are associated with disparities in access to health care.^19^

Malnutrition increases the likelihood of contracting childhood infectious diseases, including ARI.^20^, ^21^ Furthermore, malnutrition independently causes childhood mortality and morbidity.^22^ Approximately 47% of Malawian children are chronically malnourished, but there have been no studies on acute malnutrition in Malawi.^23^, ^24^ Additionally, no studies have examined malnutrition in the context of ARI in Malawi.

Thus, ARIs are a leading cause of childhood mortality and morbidity, but there is limited data on LRTI in Malawi. Prevalence studies reflect the burden of diseases on healthcare services. Therefore, this study, which aimed to establish baseline levels of disease in a rural area, will assist in planning future services and monitoring the prevalence of ARI. Furthermore, this study considered risk factors for ARI including malnutrition, PCV vaccination, number of siblings, socio‐economic status and access to health care to identify areas that preventative measures can target.

### 1.1 Aims and objectives

The primary objective of this study was to determine the annual prevalence of ARI in children aged 0‐59 months in Monkey Bay, Mangochi District, Malawi. Secondary objectives were to determine the point period prevalence of ARI and risk factors for ARI in this population.

## 2 MATERIALS AND METHODS

### 2.1 Study design

This was a cross‐sectional, population‐based study of a random sample of children aged 0‐59 months in rural Monkey Bay. Data were obtained in three ways: an oral survey, physical examination and inspection of health passports.

### 2.2 Setting and population

Malawi is divided into 28 administrative districts. Mangochi district, with an estimated population of 600 000, has lower income and poorer health than average.^25^ Mangochi is divided into five healthcare zones, one of which is Monkey Bay. Our study population included all children aged 0‐59 months living in rural Monkey Bay. Within areas, health surveillance assistants (HSAs) provide basic health assessments^26^ and refer children with symptoms of respiratory disease to healthcare providers.

Communities were defined as the area under one HSA. Some HSAs oversee multiple villages. Rural communities, with an estimated population of less than 2000 and no trading post, were eligible for inclusion. Within a community, all children aged 0‐59 months were eligible for inclusion in the study provided their parent/guardian consented and the household was registered on a pre‐study census conducted in November 2014 as part of a larger study, ensuring that participants were residents of communities sampled. Villages with a population exceeding 2000 were excluded as larger, urban areas tend to have a more transient population, and it would have therefore have been difficult to follow residents of the November 2014 census up.

### 2.3 Sampling

In the absence of prior similar studies, a conservative pre‐study sample size was calculated utilizing Raosoft (Vovici, Seattle, Washington, USA) assuming a 50% prevalence of infection and population size of 20 000. 754 children were required to detect the prevalence of LRTI with 5% precision at the 95% confidence level.

A list of the 72 HSAs in Monkey Bay was obtained. Five HSAs were excluded for having communities with a trading post. 17 HSAs were excluded for overseeing an estimated population exceeding 2000, leaving 50 HSAs eligible for selection. Six HSAs were selected using a random number generator from an alphabetical list. These oversaw eight villages. Within villages, all children aged 0‐59 months were sampled. Households were notified in advance of the researchers’ attendance and the purpose of the study by HSAs. Children were brought to a central location by their parent, and residence in the village was confirmed. After all children had been seen, households in the community were visited door‐to‐door, enabling all resident children to participate. Villages were visited on at least two separate occasions to ensure that children absent on the first visit were sampled.

### 2.4 Procedure

The purpose and procedures of the study were explained to local health authorities and permission obtained from village chiefs, aided by a trained translator. Following parental consent to participate, children were allocated a unique identification number under which data were entered.

A verbal survey, obtained from participants’ parents and facilitated by a translator, elicited data on risk factors including maternal educational level (as a proxy for socio‐economic status), clustering of disease (another child with ARI recorded in their health passport in the last 12 months in the household) and number of rooms in household and number of household residents (household density).

To determine the annual prevalence of ARI, health passports were examined. These are patient‐held records of all consultations with healthcare professionals. A positive diagnosis of ARI was recorded if an acute respiratory tract infection with clinical signs of pneumonia treated with antibiotics, lower respiratory tract Infection, or pneumonia was documented in the passport in the 12 months prior to the date of visit or since birth in infants less than a year old. Children's vaccination records, contained within the passport, were also inspected. Whether children had received all age‐appropriate PCV vaccinations and EPI mandated vaccinations for their age (as a proxy for access to health care) were recorded. In older health passports, before the addition of PCV to EPI, data on PCV status were not included in analysis as it was not possible to definitively ascertain whether or not they had received vaccination.

Assenting children were then examined physically to determine the point prevalence of ARI using integrated management of childhood illness (IMCI) guidelines by doctors/medical students from the United Kingdom who had been trained in assessing children using IMCI.^27^ Parents were asked whether their child currently had a cough and about the presence of IMCI general danger signs. The child's temperature was taken. Children's chests were then exposed and evidence of increased work of breathing (indrawing or subcostal recessions) observed. Next, respiratory rate was counted for one minute during which time the researcher listened for stridor. A positive clinical diagnosis of ARI was recorded if a child had a cough and tachypnoea (respiratory rate >50/minute in children <12 months old, respiratory rate >40/minute in children >12 months old) or cough and chest indrawing or stridor when calm.

Finally, children were assessed for malnutrition following Integrated Management of Childhood Illness Guidelines.^27^ Participants with pitting oedema present for more than two‐seconds after the researcher pressed their thumb inferior to the medial malleolus for three‐seconds were classified as acutely malnourished. Weight was measured to the nearest 0.02 Kg, with shoes and outer clothes removed, using scales that were calibrated each day. Participants aged 0‐23 months had length measured to the nearest 0.1 cm using a length board. Participants aged 24‐59 months had height measured to the nearest 0.1 cm using a vertical Leicester Height Measure®. All measurements were taken three times, with the median measure used. Measurements were then plotted on Weight‐for‐Height (acute malnutrition), Height‐for‐Age (chronic malnutrition) and Weight‐for‐Age (acute‐on‐chronic malnutrition) z‐score growth charts. Measurements plotted below ‐2 standard deviations from normal were classed as malnourished.^27^

### 2.5 Data handling

Data were entered into Microsoft Excel (Microsoft, Redmond, Washington, USA) on a tablet device at the time of gathering. Data were then checked, coded and entered into spss version 22 (IBM, New York, USA) for analysis.

### 2.6 Statistical analysis

Demographic data were first analysed. Continuous variables were tested for normality. Mean and standard deviations (S.D.) were calculated for normally distributed variables. Median and interquartile ranges (IQR) were analysed for nonparametric variables. The percentage of participants with each categorical variable was calculated.

The annual prevalence of ARI was the percentage of children with one or more episodes of ARI recorded in their health passport. The point prevalence of ARI was the percentage of children classified with clinical ARI following physical examination. For all prevalence data, 95% confidence intervals (CI) were calculated.

Univariate analysis determined potential predictor variables for current and annual ARI. Chi‐square tests examined the significance of differences between groups. Mann‐Whitney U tests examined the association between age and ARI. Variables demonstrating significance at the P < 0.10 level were entered into binary logistic regression models, for annual prevalence of ARI and point prevalence of ARI, to identify independent risk factors.

### 2.7 Ethical considerations

Ethical approval for this study was obtained from University of Birmingham BMedSci Population Sciences and Humanities Internal Ethics Review Committee (reference no. 2014‐15/CI/LJ/04), London School of Hygiene and Tropical Medicine (reference no. 6500) and Malawi College of Medicine Research Ethics Committee (reference no. P.02/14/1521).

## 3 RESULTS

### 3.1 Socio‐demographics

828 children participated. The mean size of village sampled was 156 (range 90‐300) households. An average of 67% (range 48%‐86%) of children registered on the census were found to be resident in the communities at the time of study and were sampled. Within communities, parental consent was obtained for all sampled children to participate. No data were withdrawn. Demographic information is presented in Table 1. The median (IQR) age of children was 27 (13‐41) months. 62.2% (517/828) were male. Most (53.1%, 440/828) mothers had primary‐level education. Only two mothers (0.4%) had attended further education. Most households had two adults (80.4%, 666/828) and two rooms (40.2%, 333/828). The majority of children (70.8%, 587/828) lived in households with four or fewer children. Average household density was 2.77. Vaccination records seen for 74% (613/828) of children; 73 children had lost health passports and 142 children had damaged health passports without the vaccination record. Age‐appropriate EPI vaccinations had been received in 77.2% (412/613) of children.

**Table 1:** Socio‐demographic characteristics of the included children

Socio‐demographic Variables	Number of Participants (n = 828) a
Age (Months)	27 (13‐41)
Gender	
Male	517 (62.2)
Female	311 (37.8)
Maternal Education	
None	321 (38.7)
Primary Level	440 (53.1)
Second Level	65 (7.8)
Further Education	2 (0.4)
Number of Children in Household	
1	113 (13.6)
2	178 (21.5)
3	135 (16.3)
4	161 (19.4)
5	112 (13.5)
6	62 (7.5)
7	29 (3.5)
8	22 (2.7)
9	12 (1.5)
10	4 (0.5)
Number of Adults in Household	
1	35 (4.2)
2	666 (80.4)
3	73 (8.8)
4	32 (3.9)
>4	22 (2.7)
Number of Rooms in Household	
1	224 (27.1)
2	333 (40.2)
3	216 (26.1)
4	51 (6.2)
5	4 (0.4)
Household Density	2.77 (2.03–4.38)

a Data are median [interquartile range (IQR)]; or n (%).

### 3.2 Prevalence of ARI

Health passports were available for 91.2% (755/828) of participants. The annual prevalence of ARI was 32.6% (246/755, 95% CI 29.3%‐36.0%, Standard Error (S.E.) 1.7). A sensitivity analysis, assuming that all children with missing passports did/did not have ARI, estimated the annual prevalence of ARI at 29.7%‐38.5% (95% CI 26.6%‐41.8%).

All children assented to examination. The point prevalence of ARI was 8.3% (69/828, 95% CI 6.8%‐10.4%, S.E. 0.9). 43% (356/828) of children had a cough. Of these children, 1.4% (5/356) had evidence of respiratory distress consistent with a diagnosis of severe disease. 18.3% (65/356) had tachypnoea resulting in a positive diagnosis of clinical none‐severe ARI.

### 3.3 Malnutrition

Heights were obtained for all children. Median (IQR) height was 83.3 (71.9‐91.83) cm. Weights were obtained for 825 children; three children did not assent to weighing. Mean (S.D.) weight of children was 11.09 (3.22) Kg. Acute malnutrition affected 5.3% (44/825, 95% CI 3.8%‐6.8%, (S.E.) 0.8) of children. Chronic malnutrition was present in 32.6% (270/828, 95% CI 29.5%‐35.9%, S.E. 1.6) of children. Acute‐on‐chronic malnutrition was present in 16.0% (132/825, 95% CI 13.2%‐18%, S.E. 1.3) of children.

### 3.4 Risk factors for ARI

PCV vaccination status was included in 72.0% (596/828) of health passports vaccination records; 13 children had older health passports without record of PCV. 80.0% (476/596) of children were eligible for PCV; 20.1% (120/596) had completed vaccinations prior to inclusion of PCV. PCV had been completed by 76.7% (457/476) of eligible children. The remaining 3.2% (19/476) were not vaccinated despite being eligible.

To determine risk factors associated with ARI, the proportion of children with each potential risk factor were compared in ARI positive and negative groups (see Table 2). Factors associated with increased risk of ARI in the last year included male gender (OR 3.45, 95% CI 2.49‐4.77, *P* < .01), lower socio‐economic status (*P* = .02), >four children in household (OR 1.41, 95% CI 1.01‐2.01, *P* = .05), having an effected sibling (OR 1.75, 95% CI 1.19‐2.56, *P* = .01) and acute malnutrition (OR 1.66, 95% CI 0.87‐3.14, *P* = .10). Not PCV (OR 0.62, 95% CI 0.34‐0.99, *P* = .03) and inadequate access to health care (OR 0.68, 95% CI 0.41‐0.95, *P* = .04) were protective factors. Factors significantly associated with having ARI on examination were having three or more adults in the household (OR 1.46, 95% CI 0.78‐2.60, *P* = .02), having a sibling with ARI (OR 2.09, 95% CI 1.20‐3.63, *P* < .01), acute‐on‐chronic malnutrition (OR 1.98, 95% CI 1.12‐3.50, *P* = .02) and acute malnutrition (OR 2.62, 95% CI 1.17‐5.89, *P* = .02.

**Table 2:** Risk Factors for ARI

		Point Prevalence of ARI (n = 828^a^)				Period Prevalence of ARI (n = 755^b^)			
		ARI Positive (%)^c^	ARI Negative (%)^c^	Odds Ratio (95% Confidence Interval)	*P* value^c^	ARI Positive (%)^c^	ARI Negative (%)^c^	Odds Ratio (95% Confidence Interval)	*P* value^c^
Median (Interquartile Range) Age		26 (12‐36)	27 (14‐42)	N/A	.68	27 (14‐40)	26 (13‐42)	N/A	.76
Gender	Male	45 (65.2)	470 (61.9)	1.15 (0.69‐1.93)	.61	172 (69.9)	205 (40.3)	3.45 (2.49 – 4.77)	<.01
	Female	24 (34.8)	289 (38.1)			74 (30.1)	304 (59.7)		
d(Maternal Education)	None	28 (40.6)	293 (38.6)	1.00	.65	79 (32.2)	211 (41.5)	1.00	.22
	Primary	37 (53.6)	403 (53.1)	1.04 (0.62‐1.74)	.88	139 (56.6)	266 (52.3)	1.39 (1.00‐1.94)	.05
	Second	4 (5.8)	61 (8.0)	1.46 (0.49‐4.31)	.50	27 (11.0)	31 (6.0)	2.32 (1.30‐4.14)	<.01
	Further	0 (0.0)	2 (0.3)	N/A	N/A	1 (0.2)	1 (0.2)	2.67 (0.16‐43.16)	0.49
Number of Children in Household	1‐4	51 (6.2)	536 (64.7)	1.18 (0.52 – 2.02)	.39	187 (76.0)	351 (69.1)	1.41 (1.01‐2.01)	.05
	≥5	18 (2.1)	223 (27.0)			59 (24.0)	157 (30.9)		
Number of Adults in Household	1‐2	55 (79.7)	646 (85.1)	1.46 (0.78–2.60)	.02	208 (84.6)	438 (86.1)	1.12 (0.73–1.72)	.59
	≥3	14 (20.3)	113 (14.9)			38 (15.4)	71 (13.9)		
Number of Rooms in Household	1	23 (33.3)	201 (26.5)	N/A	.59	64 (26.0)	147 (19.5)	N/A	.66
	**2**	21 (30.4)	312 (41.1)			101 (41.1)	198 (38.9)		
	**3**	21 (30.4)	195 (25.7)			67 (27.2)	130 (25.5)		
	**4**	4 (5.9)	47 (6.2)			14 (5.7)	31 (6.1)		
	**5**	0 (0.0)	4 (0.5)			0 (0.0)	3 (0.5)		
Sibling with ARI	Yes	20 (29.0)	124 (16.3)	2.09 (1.20 ‐ 3.63)	7.01(<.01**)**	57 (23.2)	75 (14.7)	1.75 (1.19 – 2.56)	<.01
	**No**	49 (71.0)	635 (83.7)			189 (76.8)	434 (85.3)		
PCV Vaccination status	Absent	14 (25.5)	124 (22.9)	1.12 (0.61‐ 2.17)	0.19 (.67)	39 (18.2)	100 (14.7)		.03
	Present	41 (74.5)	417 (22.9)			175 (81.8)	282 (85.3)		
Access to Health care (EPI)	No	23 (33.8)	177 (32.4)	1.06 (0.62 – 1.81)	0.24 (.82)	57 (27.1)	142 (35.3)	0.68 (0.41 – 0.95)	.03
	Yes	45 (66.2)	369 (67.6)			153 (72.9)	260 (64.7)		
Acute chronic Malnutrition	Yes	18 (26.1)	114 (15.1)	1.98(1.12 – 3.50)	5.78 (.02)	40 (16.2)	78 (15.3)	1.07(0.70 – 1.62)	.74
	No	51 (73.9)	642 (84.1)			206 (83.8)	431 (84.7)		
Chronic Malnutrition	Yes	26 (37.7)	244 (32.1)	1.27 (0.77 – 2.13)	1.35 (.35)	84 (34.1)	168 (33.0)	1.05 (0.76 – 1.45)	.76
	No	43 (62.3)	515 (67.8)			162 (65.9)	341 (77.0)		
Acute Malnutrition	Yes	8 (13.1)	36 (4.4)	2.62(1.17 – 5.89)	5.85 (.02)	18 (7.3)	23 (4.5)	1.66 (0.87 – 3.14)	.10
	No	61 (88.4)	720 (87.2)			228 (92.7)	486 (95.5)		

a Except for EPI data (n = 613), PCV status (n = 596), and acute malnutrition (n = 825).

b Except for EPI data (n = 613), PCV status (n = 596).

c Except for age which is presented as median (interquartile range) followed by Mann‐Whitney U (*P* Value).

d Data for primary, secondary and further level education are compared to mothers with no education (which therefore have an odds ratio of 1.00).

Forward stepwise binary logistic regression models examined factors associated with ARI when confounders such as community of residence were considered (see Tables 3 and 4). The logistic regression model for annual prevalence of ARI accounted for 16% (Nagelkerke *R* ^2^=.16) of the variance in prevalence. Having a sibling with ARI (OR 1.44, 95% CI 1.12‐1.65, *P* = .01), increasing household density (OR 2.17, 95% CI 1.82‐2.68, *P* = .02) and being acutely malnourished (OR 1.69, 95% CI 1.28‐1.76, *P* = .01) significantly increased the likelihood of having ARI.

**Table 3:** Binary Logistic Regression for the prediction of ARI in the past 12 months, identified from health passports

Predictors of ARI Infection in last 12 months		95% Confidence Interval		
Included	OR	Lower	Upper	*P* Value
Male Gender	1.21	0.83	1.47	.24
Maternal Education:				
No education to 1st level	1.24	0.83	1.86	.30
No education to 2nd level	1.32	0.63	2.77	.46
No education to 3rd level	2.35	0.13	41.25	.56
Household Density	2.17	1.82	2.68	.02
Sibling with ARI	1.44	1.12	1.65	.01
PCV Vaccination	0.91	0.48	1.75	.70
Access to Healthcare	0.75	0.42	1.35	.51
Acute Malnutrition	1.69	1.28	1.76	.01

n = 596, Cox & Snell 0.10, Nagelkerke *R* ^2^=.163, Chi‐Square 62.6, *P* < .001.

Other non‐significant factors entered into the model were age and community of residence.

**Table 4:** Binary logistic regression for the prediction of current clinical ARI, identified on examination

Predictors of Current ARI Infection		95% Confidence Interval		
Included	OR	Lower	Upper	*P* Value
Household Density	1.19	0.82	1.54	.05
Sibling with ARI	2.30	1.13	4.67	.02
Acute‐on‐chronic Malnutrition	3.06	1.23	7.61	.02
Acute Malnutrition	1.31	0.45	3.83	.63

n = 825, Cox & Snell 0.083, Nagelkerke *R* ^2^=.182, chi‐square 50.8, *P* < .001.

Other non‐significant factors entered into the model were age and community of residence.

The model produced for point prevalence of ARI accounted for 18% (Nagelkerke *R* ^2^=.18) of the variance in prevalence. Compared to those without ARI, children with ARI on examination were significantly (*P* < .05) more likely to have a sibling with ARI (OR 2.30, 95% CI 1.13–4.67, *P* = .02), live in a more densely populated household (OR 1.19, 95% CI 0.82‐1.54, *P* = .05) and have acute‐on‐chronic malnutrition (OR 3.06, 95% CI 1.23‐7.61, *P* = .02.

## 4 DISCUSSION

The only prior data on ARI in the community in Malawi estimated that 70% of children under 5 were affected.^4^ This study found that almost a third of children seek treatment for respiratory tract infections annually, suggesting a higher burden of disease than hospital estimates.^5^, ^6^ Furthermore, our study might underestimate the annual prevalence if not all affected children access health care or if there is variation in documentation in health passports. This reflects data on severe pneumonia in Malawi in which 24% of cases were identified by HSAs in the community, not in hospitals or health centres.^14^

The high point prevalence of ARI is incongruent with the annual prevalence detected. Some children might not seek medical advice meaning health passports under‐represent the level of disease. Therefore, improved access to health care in rural communities might be required. Alternatively, repeated infections of the same children and a high incidence of ARI could explain the discrepancy. The subjective diagnosis of ARI in this study may overestimate the annual prevalence. Respiratory rate is a reliable form of respiratory assessment.^28^ However, examination of children in the field was in suboptimal conditions; the presence of unfamiliar researchers could cause anxiety and subsequent tachypnoea.^29^

Univariate analysis corroborated findings from other studies that increasing household density and lower education status (annual prevalence data) increase ARI risk.^9^, ^10^, ^11^ Most households were from lower socio‐economic groups. Therefore, there may have been insufficient households in higher socio‐economic groups to detect a difference. Similarly, smaller dwellings were not associated with ARI in this study.^11^ Most houses were single‐ or double‐roomed; there might have been insufficient numbers of larger dwellings to detect a difference.

Clustering of infectious diseases is well documented.^30^ Clustering of ARI within households was the only risk factor significantly associated with both point and annual prevalence of ARI; improved sanitation may reduce disease transmission within families. Community of residence was not a significant confounder. This is likely due to geographical, environmental and social similarities between communities resulting in similar risk factors being present.

Not being vaccinated against PCV or having EPI vaccinations were protective factors for pneumonia, although this was not statistically significant, and coverage of PCV in this study was higher than estimates in neighbouring districts.^14^ It is expected that PCV vaccination protects against severe ARI. However, children with better access to health care may be more likely to have PCV and EPI vaccinations. These might be more likely to seek medical attention for ARI and have a diagnosis recorded in their health passport.

This study detected a lower prevalence of malnutrition than prior estimates.^4^, ^23^, ^24^ This could be a result school feeding programmes or seasonal variations in malnutrition; examination was undertaken after harvest; the prevalence is likely affected by the proximity to harvest season and the quality of the harvest. Acute malnutrition (examination and passport data) and acute‐on‐chronic malnutrition (examination data) were associated with ARI, but chronic malnutrition was not. Systemic inflammatory responses during acute malnutrition might increase susceptibility to infection, but this phase has passed in chronically malnourished children.^31^ Alternatively, ARI may cause children to become acutely malnourished.

The finding that males are more likely to be affected annually has been demonstrated previously. More males were sampled than females. There may have been more male children or parents may have preferentially identified male children. Similarly, bias may be present if the higher rate of ARI in males’ health passports is due to male preponderance, although the true prevalence of disease is the same especially as there was no significant difference between genders on logistic regression.

### 4.1 Strengths and limitations

The random sampling method and the fact that all sampled children participated should make results generalizable to Monkey Bay. Approximately 90% of Malawi's population lives rurally,^32^ and this study provides novel data on the burden of ARI in a rural area.

The cross‐sectional study design has limitations. Data were collected during the rainy season, and pneumonia cases peak from January to March,^14^, ^33^ possibly inflating the point prevalence of ARI. The period prevalence should not be affected. Similarly, flooding could have increased the prevalence of infectious diseases. Furthermore, temporal relationships between risk factors and ARI cannot be established; longitudinal studies are required.

Retrospectively gathering data from health passports introduces inaccuracies. Recorder bias may be present, as the detail of records varied and the diagnostic threshold may differ between practitioners. Pneumonia diagnoses may be particularly over‐recorded, in cases where a simple cough can be over‐treated with antibiotics in settings where auscultation or radiological evidence is not available. For this reason, ARI, which encompasses more severe respiratory diseases like pneumonia and simple viral infections, were considered in this study. Furthermore, disparities in access to health care may bias findings if those who are unable to access health care do not have diagnoses recorded despite being affected. This study defined the period prevalence of ARI as one or more episodes of ARI in the past year. Some children had several episodes of ARI. Considering the incidence of ARI would be useful in future studies if repeated infections cause a higher burden of disease.

Risk factors identified in this study for point prevalence of ARI had wide confidence intervals. This may be due to the small number of cases of ARI (*n *=* *69) being examined. Therefore, studies with more affected participants are required. Furthermore, risk factors entered into logistic regression models were poor predictors for ARI (Nagelkerke R^2^ 0.18 for point prevalence and 0.16 for period prevalence). Therefore, further research is required to confirm the aetiology of infection. HIV coinfection is a potential confounder^12^, ^13^ but HIV status was not considered in this study. Although approximately 10% of Malawi's population is HIV positive, in rural areas HIV status is poorly documented. Furthermore, determining HIV status in the field was beyond the scope of this study.

Many parents were uncertain of their child's age, and date of birth was often unrecorded in health passports. This problematized the calculation of malnutrition and the inclusion of older children in the study. In such cases, an estimate of age was made based on parental recollections and ages of siblings. Children were included in the study based on a pre‐study census. However, parents were sometimes unaware of the child's full name or head of household's name, problematizing identification of suitable children. Furthermore, in fishing villages there is a transient population. All available children were sampled, but in some cases, less than half of households registered on the census were occupied. In other villages, there were more households present than registered. This made it difficult to ascertain the true size of villages and the proportion of eligible children included.

### 4.2 Recommendations for future research

This study considered a remote population, which may have poorer health; further research is required to ascertain the generalizability of these findings to all Malawian children. Prospective longitudinal research could confirm the aetiology of ARI and ascertain temporal relationships between risk factors, especially malnutrition, and infection. Studies diagnosing ARI with more specific methods such as auscultation of the chest or pulse oximetry could obtain a more accurate estimate of disease prevalence as well improving accuracy for HSAs in diagnosing severe respiratory disease.

### 4.3 Conclusion

This study was the first to examine the prevalence of and risk factors for ARI in the community in Malawi. The study identified high levels of disease; 32.6% of children required treatment annually. Therefore, childhood respiratory tract infection presents a substantial burden of disease to healthcare services in rural Malawi. More children may be affected than receive treatment. Therefore, improved accesses to diagnostic and treatment facilities as well as improved primary prevention of infection are needed in this population. Malnutrition increased the likelihood of ARI. Therefore, improved nutrition may reduce ARI incidence. Furthermore, this study found significant clustering of disease within households. Thus, improved sanitation could reduce transmission of ARI. This study provides baseline data against which to compare future levels of disease and the impact of future interventions.

## CONFLICT OF INTERESTS

The authors disclose no conflict of interest.

## ETHICAL STATEMENT

This work conforms with Declaration of Helsinki guidelines.
